# Long-Term Thermo-Oil Conditioning of PA66-GF25: Non-Monotonic Tensile Response Under Combined Thermal and Lubricant Exposure

**DOI:** 10.3390/polym18162008

**Published:** 2026-08-18

**Authors:** Ronald Bastovansky, Robert Kohar, Rudolf Madaj, Peter Weis

**Affiliations:** Department of Design and Mechanical Elements, University of Žilina, 010 26 Žilina, Slovakia; robert.kohar@fstroj.uniza.sk (R.K.); rudolf.madaj@fstroj.uniza.sk (R.M.); peter.weis@fstroj.uniza.sk (P.W.)

**Keywords:** PA66-GF25, glass-fibre-reinforced polyamide, thermo-oil ageing, tensile behaviour, long-term conditioning, durability

## Abstract

Polyamide 66 reinforced with 25 wt.% short glass fibres (PA66-GF25) is widely used in engineering applications requiring long-term operation under combined thermal and lubricated conditions, including polymer bearing cage applications. However, the long-term evolution of its mechanical behaviour under thermo-oil exposure remains insufficiently documented, particularly over extended exposure periods relevant for service-oriented durability assessment. This study investigates the tensile behaviour of PA66-GF25 after immersion in an industrial bearing lubricant for up to 12 months at conditioning temperatures of −30 °C, 24 °C, and 60 °C. Tensile tests were primarily performed at 24 °C to evaluate the influence of conditioning history, while selected specimens were additionally tested at 60 °C to assess the effect of testing temperature. The results indicate that conditioning temperature strongly influences the evolution of tensile behaviour. Specimens thermo-oil-conditioned at 24 °C and −30 °C exhibited a non-monotonic evolution of tensile strength, characterised by an initial reduction after 4 months followed by recovery and an apparent tendency towards stabilisation at longer exposure durations. In contrast, specimens thermo-oil-conditioned at 60 °C exhibited a continuous increase in tensile strength throughout the investigated period. Although elevated testing temperatures reduced the absolute tensile strength, the relative trends associated with thermo-oil conditioning remained observable. The findings indicate that long-term thermo-oil exposure of PA66-GF25 does not necessarily lead to continuous degradation of tensile performance under the investigated conditions. Instead, the material exhibits a transient response at intermediate exposure durations followed by recovery or an apparent tendency towards stabilisation of tensile performance. These results provide a long-term experimental tensile dataset for PA66-GF25 under combined thermal and lubricant exposure conditions and highlight the importance of extended conditioning when evaluating material performance for lubricated engineering applications. The findings show that intermediate exposure data may not be sufficient for assessing long-term tensile behaviour. However, the mechanisms responsible for the observed tensile strength evolution require further verification using complementary physicochemical and microstructural analyses.

## 1. Introduction

Glass-fibre-reinforced polyamides are among the most widely used engineering thermoplastics due to their favourable combination of mechanical strength, stiffness, wear resistance, dimensional stability and processability. In particular, polyamide 66 reinforced with short glass fibres (PA66-GF) has found extensive application in automotive systems, industrial machinery and tribological components operating under demanding service conditions. The incorporation of glass fibres significantly improves the stiffness and tensile strength of polyamide matrices while maintaining relatively low weight and good manufacturing efficiency. Consequently, glass-fibre-reinforced PA66 is frequently considered for load-bearing components exposed to combined mechanical and environmental loading conditions [1,2,3].

The long-term performance of polyamide-based composites is strongly influenced by environmental exposure. Due to the presence of polar amide groups, polyamides exhibit affinity towards water and other polar fluids, which may affect their physical and mechanical properties. Water absorption, moisture conditioning and hygrothermal ageing have been extensively investigated and have been shown to influence stiffness, strength, ductility, dimensional stability and glass transition behaviour [3,4,5,6,7,8]. These changes are commonly attributed to fluid uptake and plasticisation phenomena occurring within the polymer matrix [3,5,7,9]. For reinforced systems, environmental exposure may also affect the overall load transfer mechanisms between the matrix and reinforcing fibres, thereby influencing the mechanical response of the composite [2,8].

Besides moisture-related ageing, polyamides are frequently exposed to various industrial fluids during service. Thomason and Porteus [10,11] demonstrated that prolonged exposure of glass-fibre-reinforced PA66 to automotive fluids may result in fluid absorption, dimensional changes and modifications of mechanical performance. Similarly, Wei et al. [9] reported changes in the behaviour of glass-fibre-reinforced polyamide composites exposed to bioethanol fuel at elevated temperatures. More recently, studies addressing lubricant compatibility of polyamides have confirmed that prolonged contact with lubricating fluids can alter the physical and mechanical characteristics of polyamide materials [12,13]. Such observations highlight the necessity of evaluating material performance under realistic service environments rather than relying solely on short-term laboratory testing.

Thermal exposure represents another important factor affecting the durability of glass-fibre-reinforced polyamides. Previous investigations have shown that elevated temperatures may induce changes in mechanical performance through physical ageing and degradation processes. Salvi et al. [14] observed that thermal ageing of glass-fibre-reinforced PA66 can initially result in temporary improvements of mechanical properties associated with annealing effects, followed by progressive deterioration during prolonged exposure. Similar observations were reported by Autay et al. [15], who investigated the influence of thermal ageing on the mechanical and tribological behaviour of short glass-fibre-reinforced PA66 composites. Previous studies have shown that ageing-induced changes in mechanical properties do not always follow a simple linear trend and may depend strongly on exposure conditions and ageing duration [3,8,14,15]. Consequently, the evolution of mechanical properties during ageing may be more complex than a simple monotonic decrease and may depend on the interaction between environmental conditions, exposure duration and material structure.

Although numerous studies have addressed thermal, hygrothermal and fluid-induced ageing of polyamides, most available investigations report exposure periods ranging from several days to approximately three months, whereas studies extending to one-year service-relevant durations remain comparatively uncommon [7,8,9,10,11,12,13,14,15]. For example, Ksouri et al. [8] investigated long-term ageing of PA6 and PA6GF30 under severe hygrothermal conditions and reported substantial changes in mechanical performance during prolonged conditioning. However, comparable information for PA66-GF materials exposed to industrial lubricants over extended periods remains limited. Furthermore, the long-term evolution of tensile behaviour under extended thermo-oil ageing remains insufficiently documented.

This knowledge gap is particularly relevant for polymer components used in lubricated engineering systems such as bearing cages, where materials are expected to operate for extended periods under simultaneous thermal and lubricant exposure. Previous studies have addressed the reliability and service-life assessment of large-scale rolling bearings using dedicated testing and diagnostic monitoring approaches, while the dynamic behaviour of bearing cages, including the influence of cage elasticity, has been investigated using numerical modelling techniques [16,17]. Reliable long-term material data are therefore essential for durability assessment and engineering design of polymer bearing cage components.

The motivation for the present testing programme is related to the development of polymer cage concepts for large-scale rolling bearings, including bearing cages intended for wind turbine applications. Conventional large bearing cages are commonly produced from steel, and their manufacture can be costly and technologically demanding due to forming or machining operations, material waste, long production times and high manufacturing costs associated with these production routes. Polymer cage materials may offer potential manufacturing and design advantages; however, their application requires verification that the material retains sufficient mechanical performance after long-term exposure to bearing lubricant and service-relevant temperatures. Such information is needed as input for service-oriented material assessment, finite element modelling and optimisation of polymer cage designs intended for long service lives.

The present study investigates the long-term thermo-oil ageing behaviour of PA66 reinforced with 25 wt.% short glass fibres (PA66-GF25) as a candidate material for polymer bearing cage applications. Specimens were immersed in an industrial bearing lubricant and conditioned for up to 12 months at temperatures of −30 °C, 24 °C and 60 °C. These conditioning temperatures were selected to represent service-relevant thermal conditions that can be reproduced under laboratory conditions. Tensile testing was subsequently performed to evaluate the evolution of mechanical behaviour as a function of conditioning temperature and exposure duration. The one-year exposure period was selected as a practically feasible long-term screening duration intended to reveal whether combined thermal and lubricant exposure leads to pronounced degradation of tensile performance.

The main contribution of this study is the provision of long-term experimental tensile data for PA66-GF25 under combined thermal and lubricant exposure conditions. Beyond reporting tensile strength values, the study demonstrates that the tensile response may be non-monotonic and strongly dependent on conditioning temperature, exposure duration and testing temperature. This is relevant for durability assessment and FEM-based evaluation of polymer cage concepts because short-term or intermediate exposure data may not be sufficient to infer long-term tensile behaviour. The underlying ageing mechanisms are not directly identified in the present study and are therefore treated as hypotheses requiring further physicochemical and microstructural verification.

Accordingly, the present work should be understood as a service-oriented long-term tensile screening study rather than as a complete mechanistic ageing investigation. Its scientific contribution lies in experimentally documenting the macroscopic tensile response of PA66-GF25 after one year of combined lubricant and thermal exposure, identifying the non-monotonic evolution of tensile strength and demonstrating that intermediate exposure durations may not be sufficient to infer longer-term tensile behaviour. The study therefore provides experimentally based evidence for material screening and further component-oriented development, while direct identification of the underlying ageing mechanisms remains a subject for future work.

## 2. Materials and Methods

### 2.1. Material

The investigated material was Ultramid^®^ A3HG5 (BASF SE, Ludwigshafen, Germany), a polyamide 66 reinforced with 25 wt.% short glass fibres (PA66-GF25). According to the manufacturer, the material is intended for injection-moulded engineering components requiring high stiffness and dimensional stability, including machinery components and bearing cages [18]. The material was supplied in the form of granules and subsequently processed into ISO 527-1A tensile specimens by injection moulding under laboratory conditions [19]. Injection moulding was performed using the processing conditions recommended by the material supplier, including a melt temperature of approximately 290 °C and a mould temperature of approximately 80 °C [18]. Selected material properties of Ultramid^®^ A3HG5 are summarised in Table 1.

As the conditioning medium, the industrial bearing lubricant Klüberplex BEM 41-141 was used [20]. This lubricant is a lithium-complex-soap-based grease formulated with mineral oil and synthetic hydrocarbon oil. According to the manufacturer, it is intended for rolling and plain bearings subjected to high loads, including rotor, generator and pitch bearings in wind turbines. The specified service temperature range is −40 °C to 150 °C. Selected technical parameters include an approximate density of 0.88 g/cm^3^ at 20 °C, NLGI grade 1, and base-oil kinematic viscosity values of approximately 130 mm^2^/s at 40 °C and 14 mm^2^/s at 100 °C [20]. The lubricant was selected to simulate service-relevant thermo-lubrication conditions encountered in highly loaded bearing applications.

### 2.2. Thermo-Oil Conditioning

Specimens were subjected to long-term thermo-oil ageing by immersion in the industrial bearing lubricant Klüberplex BEM 41-141. The conditioning procedure was designed to simulate combined thermal and lubricant exposure conditions relevant for polymer cage components operating in rolling bearing systems.

Two types of non-immersed reference specimens were used for comparison in this study. The initial dry reference specimens (0 M) were produced in the same injection-moulding batch as the specimens used for thermo-oil conditioning, were not immersed in the lubricant and were not subjected to long-term thermal conditioning. These specimens were tested shortly after specimen preparation and were used to define the initial tensile behaviour of the material. For the 0 M reference specimens, the temperature indicated in the notation refers only to the tensile testing temperature and not to a preceding ageing temperature.

It should be noted that the term “initial dry reference” denotes specimens that were not immersed in lubricant and were not subjected to long-term thermal conditioning. The specimens were tested in the initial laboratory-conditioned state and were not additionally oven-dried before tensile testing in order to represent a practical non-immersed reference condition. Their actual moisture content was not measured.

In addition, non-immersed temperature-conditioned reference specimens were stored for 4, 8 and 12 months under the same temperature conditions as the immersed specimens (−30 °C, 24 °C, and 60 °C), but without lubricant exposure. These specimens were used as non-immersed temperature-conditioned controls to distinguish the effect of long-term thermal exposure from the combined effect of thermal exposure and lubricant immersion.

The immersed specimens remained fully immersed in the lubricant throughout the entire conditioning period.

The conditioning was carried out at three temperatures: −30 °C, 24 °C, and 60 °C, representing sub-ambient, room-temperature, and elevated operating conditions, respectively. For each immersed thermo-oil conditioning temperature and exposure duration, five specimens were prepared. Conditioning was performed in sealed containers to minimise lubricant loss and contamination.

The sub-ambient conditioning (−30 °C) was performed in a laboratory freezer, room-temperature conditioning (24 °C) under ambient laboratory conditions, and elevated-temperature conditioning (60 °C) in a temperature-controlled chamber.

The temperature during sub-ambient conditioning was checked at regular intervals.

Exposure durations of 4, 8, and 12 months were investigated in order to evaluate both intermediate and long-term effects of thermo-oil ageing.

After exposure, specimens were removed from the lubricant, gently wiped using absorbent paper to remove excess lubricant from the surface, sealed in plastic bags and transported to the laboratory. Tensile testing was performed within approximately 24–48 h after removal from the conditioning medium.

### 2.3. Tensile Testing

Tensile tests were performed using a LabTest 5.20 ST universal testing machine (Labortech s.r.o., Opava, Czech Republic) with a maximum load capacity of 30 kN. The machine was calibrated according to ISO 7500-1 [21] and classified as accuracy class 0.5.

Tensile testing was performed using type 1A specimens and generally followed the procedures specified in ISO 527-1 [19]. Tensile tests were conducted under displacement control. The crosshead speed was set to 2 mm/min up to approximately 0.3% strain and subsequently increased to 5 mm/min until fracture.

The majority of specimens were tested at a nominal temperature of 24 °C in order to evaluate the effect of thermo-oil conditioning on tensile behaviour. Additional tests were performed for selected conditioned specimens at elevated (60 °C) and sub-ambient testing temperatures, as described in Section 2.5 and Section 2.6.

Strain was evaluated from crosshead displacement and was used primarily for comparative assessment of the overall stress–strain response. Tensile modulus and nominal strain-at-break values were therefore not used as primary quantitative indicators in this study. The main quantitative evaluation was based on tensile strength, which could be consistently determined for all valid specimens and testing conditions.

Because strain was derived from crosshead displacement rather than measured directly over the specimen gauge length using an extensometer, the recorded displacement may include contributions from the compliance of the testing machine, grips and loading system. Consequently, the stress–strain curves presented in this study should be interpreted as apparent engineering stress–strain responses suitable for comparative assessment between testing conditions. This limitation mainly affects the absolute strain values, strain-at-break and tensile modulus. It has a substantially smaller influence on tensile strength, which was calculated from the measured force and the initial specimen cross-sectional area. For this reason, tensile strength was selected as the primary quantitative parameter for evaluating the effect of thermo-oil conditioning.

For each condition, up to five specimens were tested. Invalid failures occurring within the clamping region were excluded from the analysis. The number of valid specimens included in the statistical evaluation is reported for each condition in Table 2. Average values of tensile strength (R_m_) were subsequently calculated from the valid specimens.

### 2.4. Measurement of Specimen Dimensions and Stress Calculation

The specimen width was measured using a calliper with an accuracy of ±0.02 mm, while the thickness was measured using a micrometer with an accuracy of ±0.01 mm. The width and thickness were measured at five locations along the gauge length and the average values were used for subsequent calculations.

The initial cross-sectional area of each specimen was determined from the measured dimensions. Engineering stress was calculated as the applied force divided by the initial cross-sectional area. The tensile strength (R_m_) was defined as the maximum engineering stress reached during the test.

All calculations were performed using the initial specimen dimensions without correction for necking or reduction in cross-section during deformation. Consequently, all reported stress values correspond to engineering stress.

### 2.5. Elevated-Temperature Testing

Prior to testing, specimens were preconditioned for approximately 2 h in a temperature-controlled chamber maintained at 60 ± 0.5 °C. The specimen temperature was verified using a contact temperature sensor immediately before testing. During tensile testing, the specimen gauge section was enclosed within an insulated cylindrical chamber heated by a controlled hot-air source (Makita HG650C, Makita Corporation, Anjo, Japan).

Continuous real-time measurement of specimen temperature was not available during testing. Consequently, the reported test temperature of 60 °C should be regarded as a nominal testing condition based on preconditioning and the applied heating arrangement. All elevated-temperature tests were performed using the same apparatus, preconditioning procedure and heating configuration to ensure comparability between measurements.

### 2.6. Sub-Ambient Testing

It should be noted that the temperatures −30 °C and 60 °C refer to long-term conditioning conditions and not to the temperature of the tensile test itself.

Sub-ambient tensile tests were performed using dry ice (solid CO_2_) in order to establish low-temperature testing conditions.

Prior to testing, specimens were cooled in dry ice for approximately 1 h. A representative specimen instrumented with a contact temperature sensor exhibited a surface temperature of approximately −60 °C immediately before testing.

During tensile testing, the specimen gauge section was enclosed within an insulated cylindrical chamber. The chamber was continuously supplied with dry ice throughout the test in order to limit specimen warming during loading.

Continuous real-time measurement of specimen temperature during testing was not available. Therefore, the reported results should be interpreted as corresponding to nominal sub-ambient testing conditions established by the applied cooling procedure.

All sub-ambient tests were performed using the same cooling arrangement and testing procedure to ensure comparability between measurements. These sub-ambient results are reported in the Supplementary Materials and were not included in the main statistical evaluation of tensile strength trends.

### 2.7. Data Evaluation and Statistical Analysis

For each testing condition, tensile strength (R_m_) values were calculated from the valid specimens and are reported as mean values ± standard deviation (SD).

Normalised tensile strength values were determined relative to the initial dry reference condition (0 M) in order to facilitate comparison of relative changes induced by thermo-oil ageing.

The evaluation focused primarily on comparative analysis of the effects of conditioning temperature and exposure duration on tensile behaviour. Due to the limited number of valid specimens available for some conditions, as reported in Table 2, formal statistical significance testing was not performed. Instead, the interpretation was based on mean values, standard deviations, coefficients of variation and the consistency of observed trends across exposure durations and conditioning temperatures. Differences between conditions were therefore interpreted cautiously, particularly when the difference between mean values was small relative to the corresponding standard deviations.

Specimens exhibiting invalid failure outside the gauge section (e.g., within the clamping region) were excluded from the evaluation in accordance with ISO 527 recommendations.

**Figure 1:**
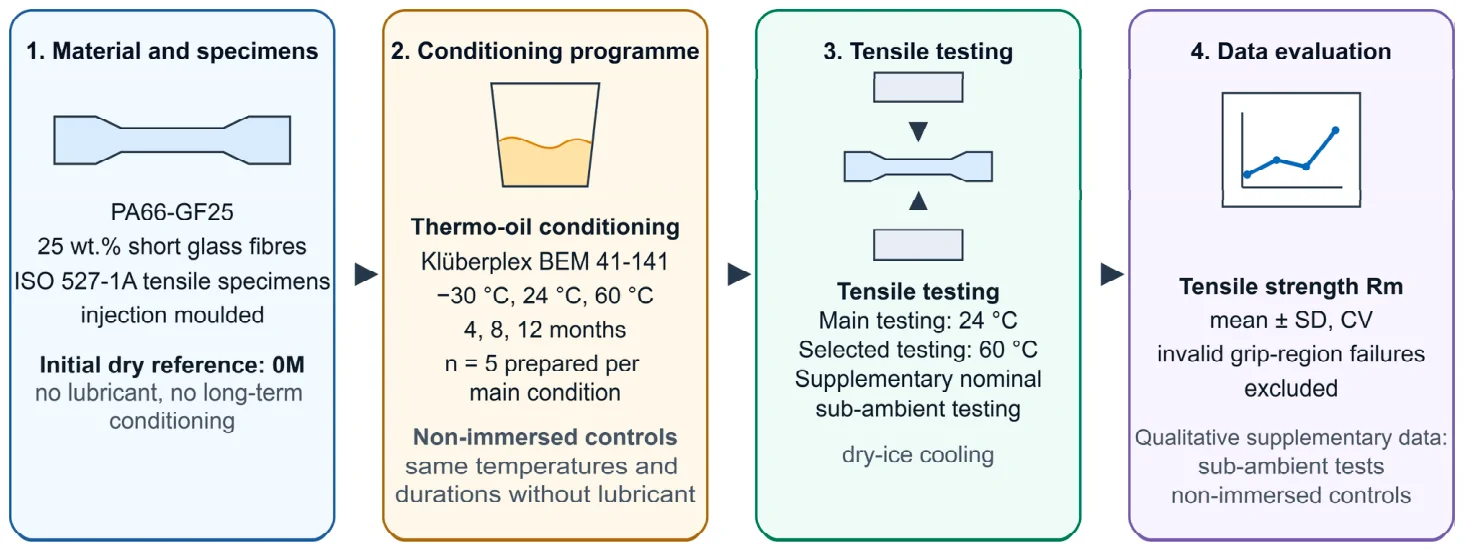
Schematic overview of the experimental programme used to evaluate the long-term thermo-oil tensile behaviour of PA66-GF25. Injection-moulded ISO 527-1A specimens were evaluated as initial dry references or subjected to thermo-oil conditioning in Klüberplex BEM 41-141 lubricant at −30 °C, 24 °C and 60 °C for 4, 8 and 12 months. The main tensile tests were performed at 24 °C, while selected specimens were tested at 60 °C and under supplementary nominal sub-ambient dry-ice cooling conditions. Non-immersed temperature-conditioned controls and nominal sub-ambient results were used as qualitative supplementary reference data.

All data processing and graphical representation were performed using Microsoft Excel for Microsoft 365 (Microsoft Corporation, Redmond, WA, USA). An overview of the experimental programme, including specimen preparation, thermo-oil conditioning, testing temperatures and data evaluation strategy, is shown in Figure 1.

**Figure 2:**
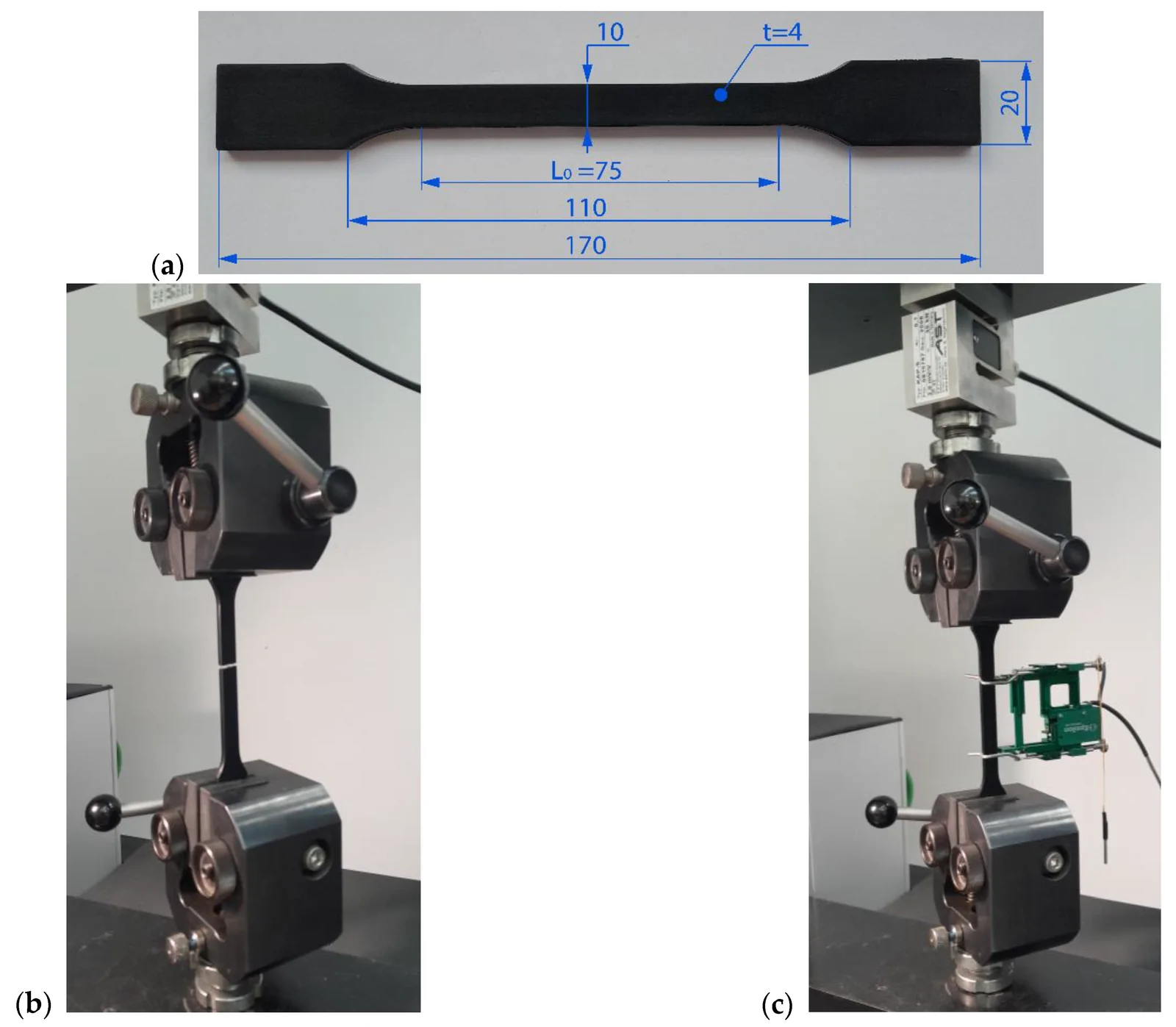

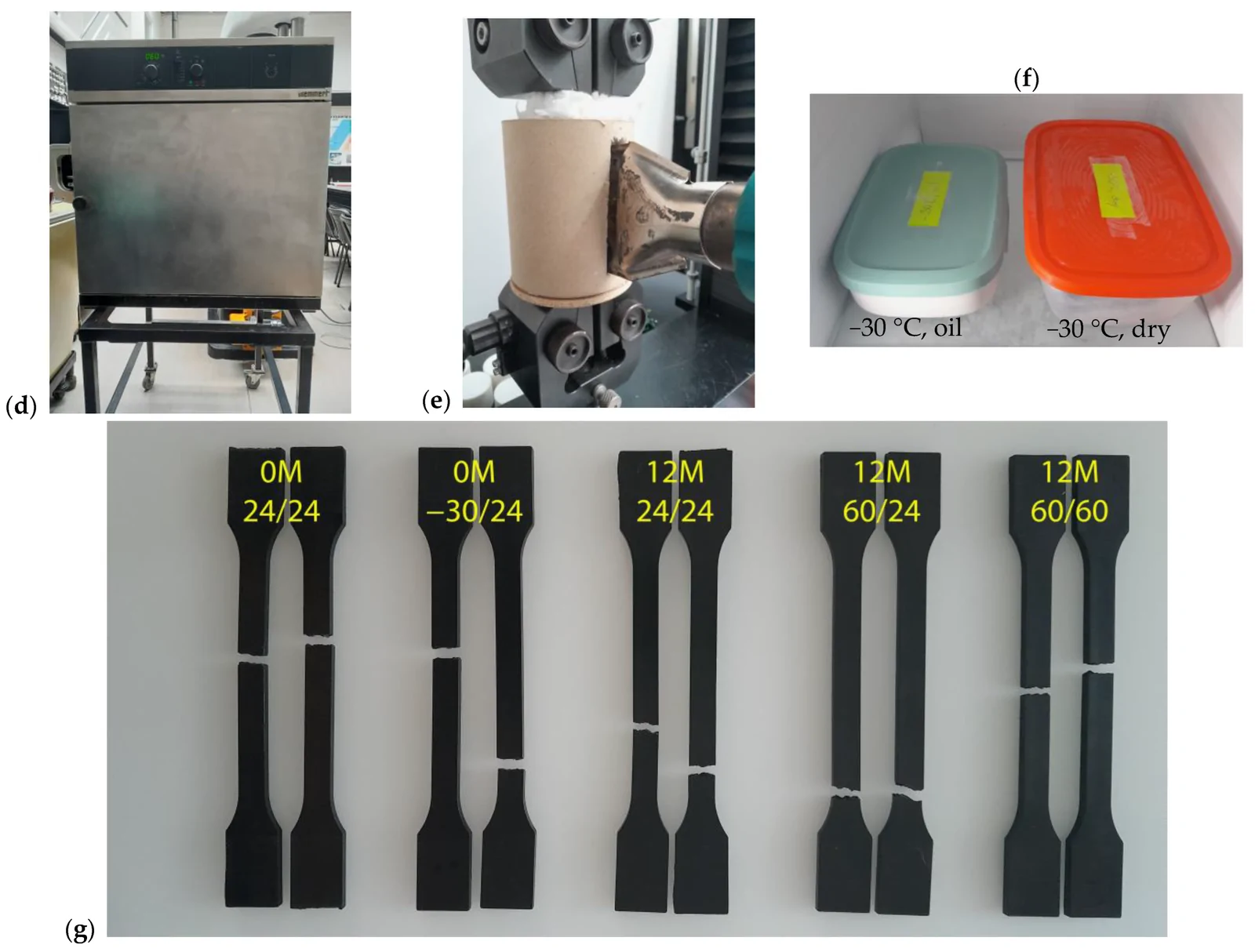
Photographic documentation of the experimental arrangements: (**a**) ISO 527-1A tensile specimen geometry with nominal dimensions in millimetres; (**b**) tensile specimen mounted in the testing machine; (**c**) representative tensile testing arrangement with clip-on extensometer; (**d**) temperature chamber used for elevated-temperature conditioning and specimen preconditioning before tensile testing; (**e**) insulated enclosure used for temperature-controlled tensile testing, shown with controlled hot-air heating for elevated-temperature tests; (**f**) low-temperature freezer used for long-term sub-ambient conditioning of sealed containers with non-immersed and thermo-oil-conditioned specimens; and (**g**) representative fractured tensile specimens after testing for selected reference and 12-month thermo-oil-conditioned conditions.

In addition to the schematic overview, photographic documentation of the specimen geometry, tensile testing arrangements, temperature-conditioning equipment and representative fractured specimens is provided in Figure 2. The figure was included to improve the transparency and reproducibility of the experimental programme and to document the actual testing and conditioning arrangements used in the study.

## 3. Results

The tensile strength values obtained for the initial dry reference specimens and thermo-oil-conditioned specimens are summarised in Table 2. The notation A/B denotes conditioning temperature (A) and testing temperature (B). For the 0 M dry reference condition, the indicated temperature refers only to the tensile testing temperature and not to a preceding ageing temperature. Non-immersed temperature-conditioned control specimens were evaluated as supporting reference data and are reported in Table S2. Since only one valid specimen was available for each non-immersed control condition, these data are used only for qualitative comparison. The results are subsequently discussed according to the testing temperature and exposure duration.

### 3.1. Tensile Strength at 24 °C After Thermo-Oil Ageing

**Figure 3:**
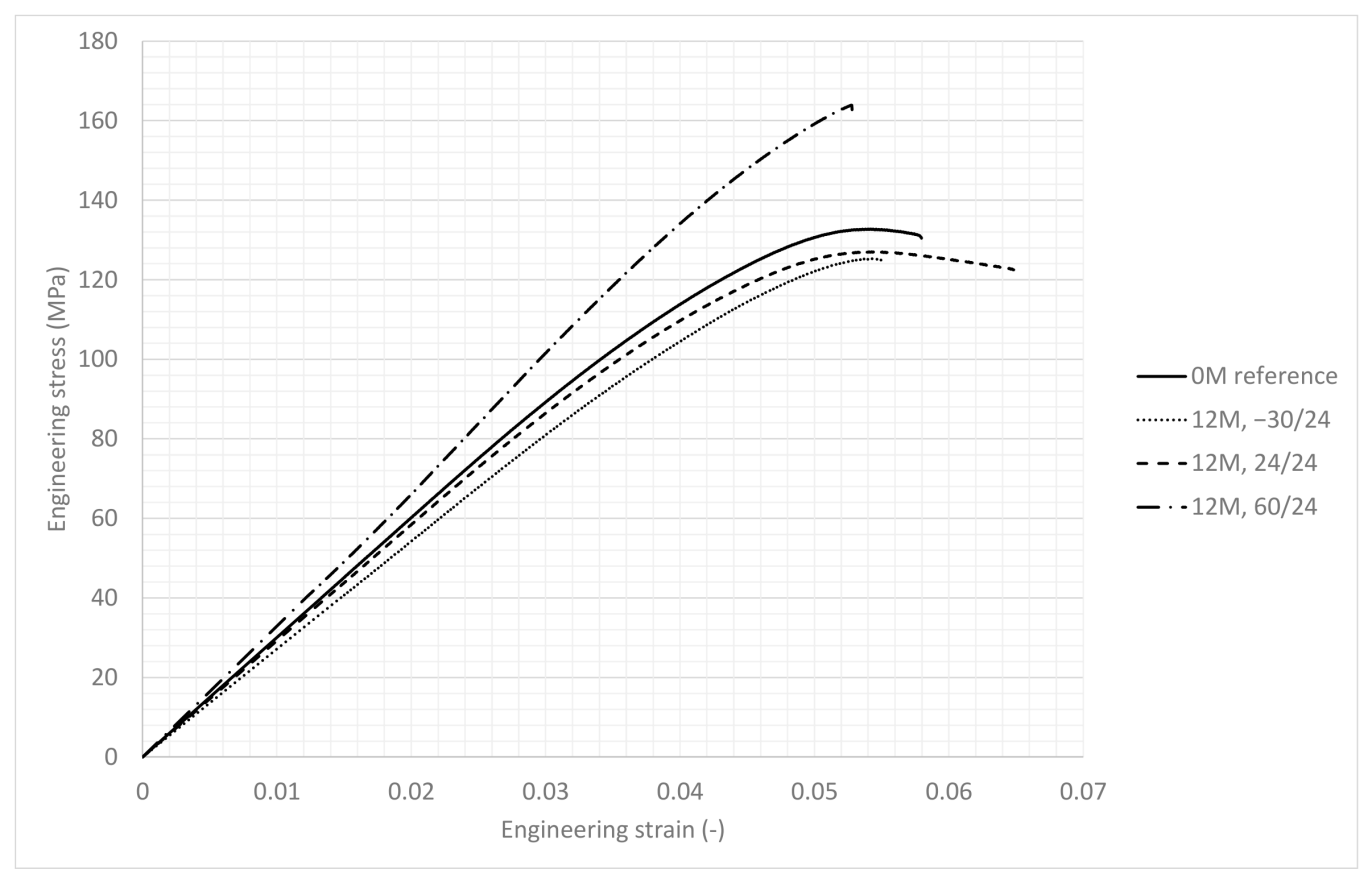
Representative engineering stress–strain curves of PA66-GF25 initial dry reference and thermo-oil-conditioned specimens tested at 24 °C. The notation A/B denotes conditioning temperature (A) and nominal testing temperature (B). For the 0 M dry reference, the indicated temperature refers only to the tensile testing temperature. Strain was derived from crosshead displacement and should therefore be interpreted as apparent engineering strain.

Representative engineering stress–strain curves obtained from specimens tested at 24 °C after thermo-oil conditioning are shown in Figure 3. The overall shape of the curves remained similar for all investigated conditions; however, differences in tensile strength were observed depending on conditioning temperature and exposure duration.

**Figure 4:**
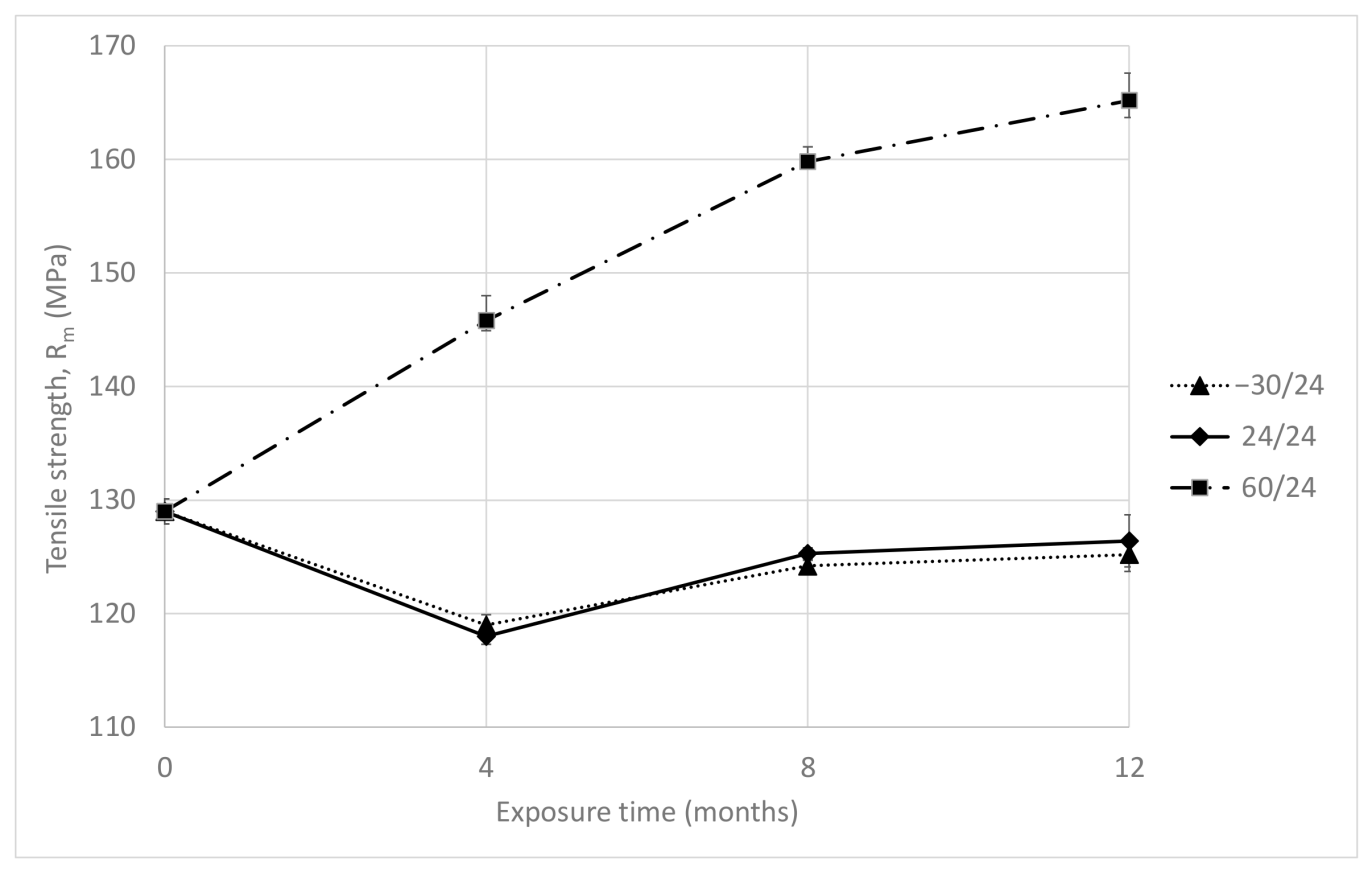
Evolution of tensile strength R_m_ of PA66-GF25 as a function of exposure time for thermo-oil-conditioned specimens tested at 24 °C. The notation A/B denotes conditioning temperature (A) and testing temperature (B). Error bars indicate standard deviation.

The evolution of tensile strength (R_m_) during thermo-oil conditioning is summarised in Table 2 and presented graphically in Figure 4. For specimens thermo-oil-conditioned at 24 °C and tested at 24 °C, tensile strength decreased from 130.3 ± 3.8 MPa for the initial dry reference condition to 118.0 ± 0.7 MPa after 4 months of exposure. Subsequently, tensile strength increased to 125.3 ± 0.5 MPa after 8 months and remained nearly unchanged after 12 months (126.4 ± 2.3 MPa). A comparable trend was observed for specimens thermo-oil-conditioned at −30 °C and tested at 24 °C, where tensile strength decreased to 119.0 ± 0.9 MPa after 4 months and then increased to 124.2 ± 0.5 MPa and 125.2 ± 1.5 MPa after 8 and 12 months, respectively.

After 8 and 12 months of thermo-oil conditioning, specimens conditioned at −30 °C and 24 °C and tested at 24 °C exhibited very similar tensile strength values, indicating convergence of the tensile response despite different conditioning temperatures.

In contrast, specimens thermo-oil-conditioned at 60 °C and tested at 24 °C exhibited a continuous increase in tensile strength throughout the investigated conditioning period. Tensile strength increased from 145.8 ± 2.2 MPa after 4 months to 159.8 ± 1.3 MPa after 8 months and reached 165.2 ± 2.4 MPa after 12 months. These values exceeded the tensile strength of the initial dry reference condition tested at 24 °C by approximately 12%, 23% and 27%, respectively, representing the highest tensile strength values obtained in the main 24 °C testing series.

The interpretation of these differences should consider the scatter of the measured data. For specimens conditioned at −30 °C and 24 °C, the decrease in tensile strength after 4 months was larger than the corresponding standard deviations and is therefore interpreted as a clear initial reduction in tensile performance. In contrast, the differences between the 8-month and 12-month values for these two conditioning temperatures were small and partly comparable with the experimental scatter. These results are therefore interpreted as recovery followed by an apparent tendency towards stabilisation rather than as a statistically verified increase between 8 and 12 months. For specimens conditioned at 60 °C, the increase in tensile strength between 4, 8 and 12 months was substantially larger than the corresponding standard deviations, indicating a clearer monotonic increasing trend under this conditioning condition.

Overall, the results indicate that the evolution of tensile strength during thermo-oil conditioning was not characterised by continuous degradation under the investigated conditions. Instead, the thermo-oil-conditioned specimens exhibited an initial decrease in tensile strength after 4 months of conditioning at −30 °C and 24 °C, followed by a gradual recovery and an apparent tendency towards stabilisation at longer exposure durations.

In contrast, thermo-oil conditioning at 60 °C resulted in a sustained increase in tensile strength throughout the investigated exposure period.

### 3.2. Comparison of Tensile Strength at 24 °C and 60 °C

In addition to conditioning history, the influence of tensile testing temperature on the measured tensile strength was evaluated. For specimens thermo-oil-conditioned at 60 °C, tensile tests were performed at both 24 °C and 60 °C, allowing the effect of elevated testing temperature to be separated from the effect of long-term thermo-oil conditioning.

**Figure 5:**
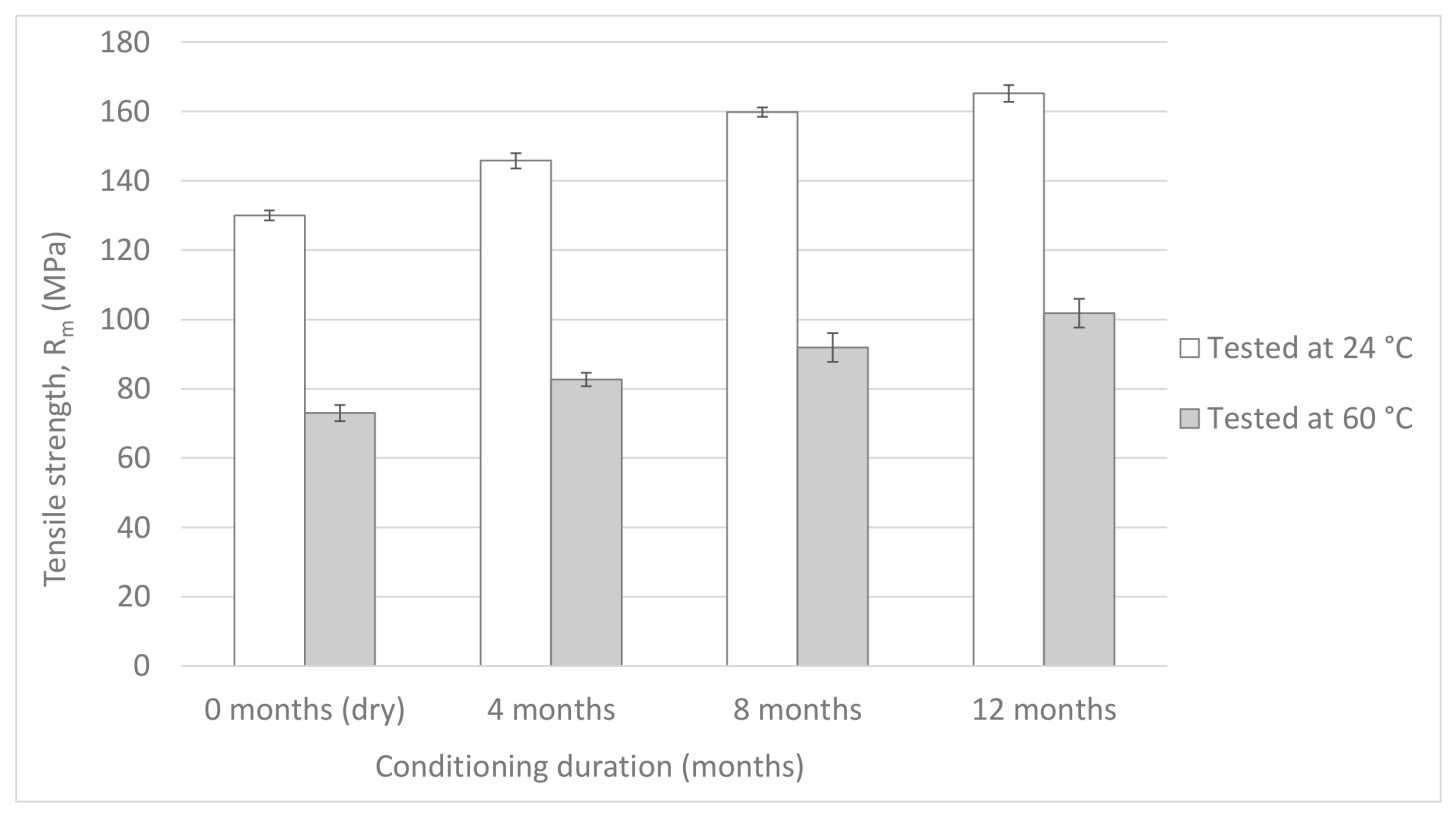
Effect of testing temperature on the tensile strength R_m_ of PA66-GF25 initial dry reference and thermo-oil-conditioned specimens. Thermo-oil-conditioned specimens were conditioned at 60 °C and subsequently tested at either 24 °C or 60 °C. Error bars indicate standard deviation.

The results are summarised in Figure 5. Increasing the testing temperature from 24 °C to 60 °C resulted in a pronounced reduction in tensile strength for all investigated exposure durations. For example, after 12 months of thermo-oil conditioning at 60 °C, tensile strength decreased from 165.2 ± 2.4 MPa when tested at 24 °C to 101.8 ± 4.1 MPa when tested at 60 °C.

Despite this reduction in absolute tensile strength at elevated testing temperatures, the effect of thermo-oil conditioning remained observable. For all exposure durations, specimens thermo-oil-conditioned at 60 °C exhibited higher tensile strength than the corresponding initial dry reference condition tested at the same temperature. Furthermore, the increase in tensile strength with increasing exposure duration was observed for specimens tested at both 24 °C and 60 °C.

These results indicate that the testing temperature and thermo-oil conditioning affect different aspects of the tensile response. Elevated testing temperature substantially reduced the measured tensile strength, whereas the relative trend associated with long-term thermo-oil conditioning remained observable under both testing temperatures.

### 3.3. Time Evolution and Apparent Stabilisation of Tensile Behaviour

The combined evaluation of tensile strength evolution with exposure time reveals different trends depending on the thermo-oil conditioning temperature. As shown in Figure 4, the tensile response of PA66-GF25 did not follow a simple monotonic degradation trend under the investigated conditions. Instead, a transient response was observed at intermediate exposure durations.

For specimens thermo-oil-conditioned at 24 °C and −30 °C and tested at 24 °C, tensile strength initially decreased after 4 months, followed by a recovery at 8 months and an apparent tendency towards stabilisation at 12 months. This observation indicates that the reduction observed after 4 months did not continue progressively with increasing exposure time.

In contrast, specimens thermo-oil-conditioned at 60 °C and tested at 24 °C exhibited a continuous increase in tensile strength with exposure time. However, the increase between 8 and 12 months was smaller than that observed between 4 and 8 months, suggesting a reduced rate of change at longer exposure durations.

**Figure 6:**
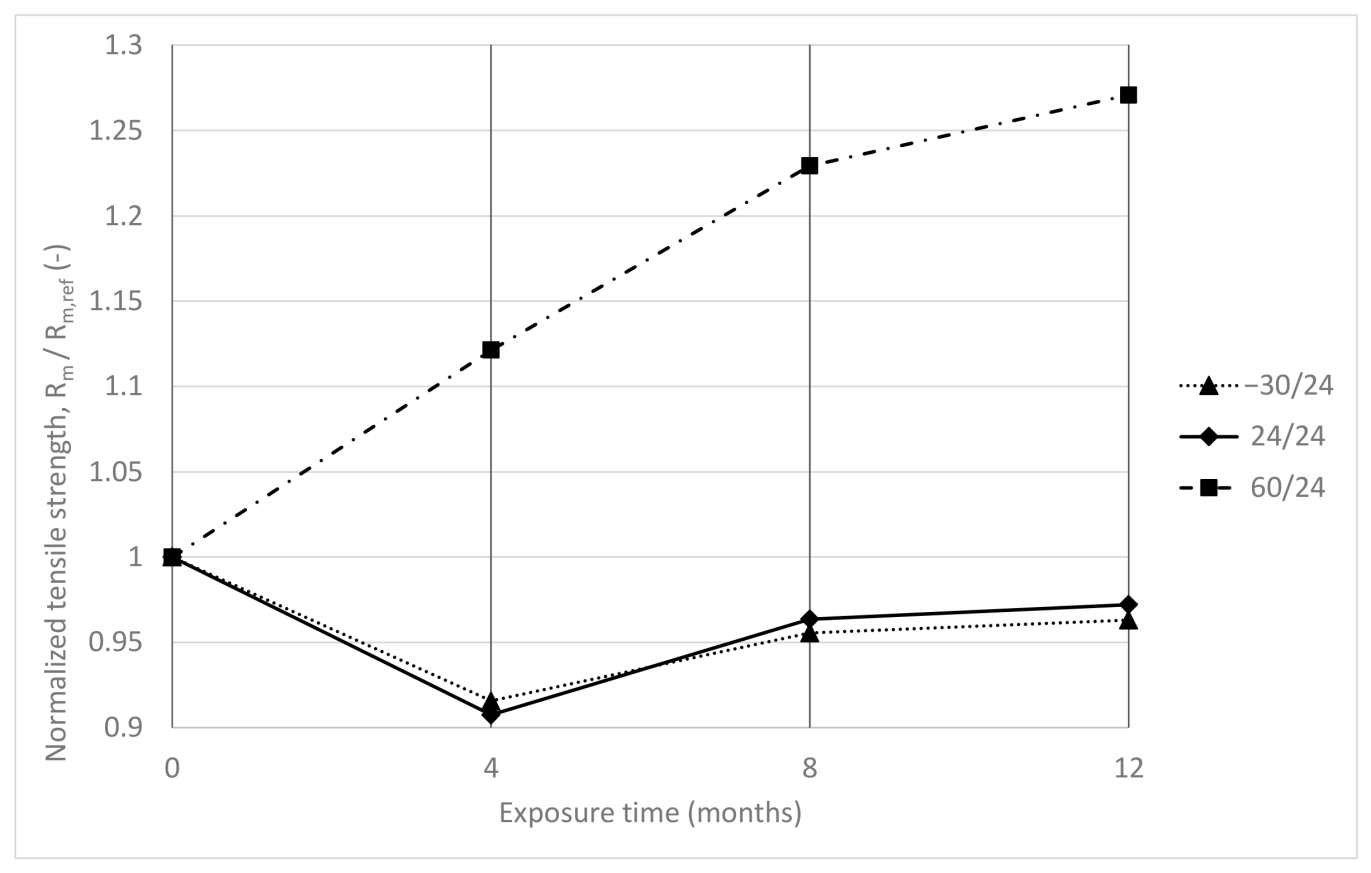
Normalised evolution of tensile strength R_m_ of PA66-GF25 as a function of exposure time for thermo-oil-conditioned specimens tested at 24 °C. Values are normalised with respect to the initial dry reference condition tested at 24 °C. The notation A/B denotes conditioning temperature (A) and testing temperature (B).

The normalised representation shown in Figure 6 highlights the different evolution of tensile strength for the investigated thermo-oil conditioning temperatures. For specimens thermo-oil-conditioned at 24 °C and −30 °C, tensile strength decreased to approximately 90–92% of the initial dry reference value after 4 months and subsequently recovered to approximately 96–97% after 12 months. In contrast, specimens thermo-oil-conditioned at 60 °C reached approximately 127% of the initial non-immersed reference strength after 12 months.

### 3.4. Representative Fracture-Surface Morphology

**Figure 7:**
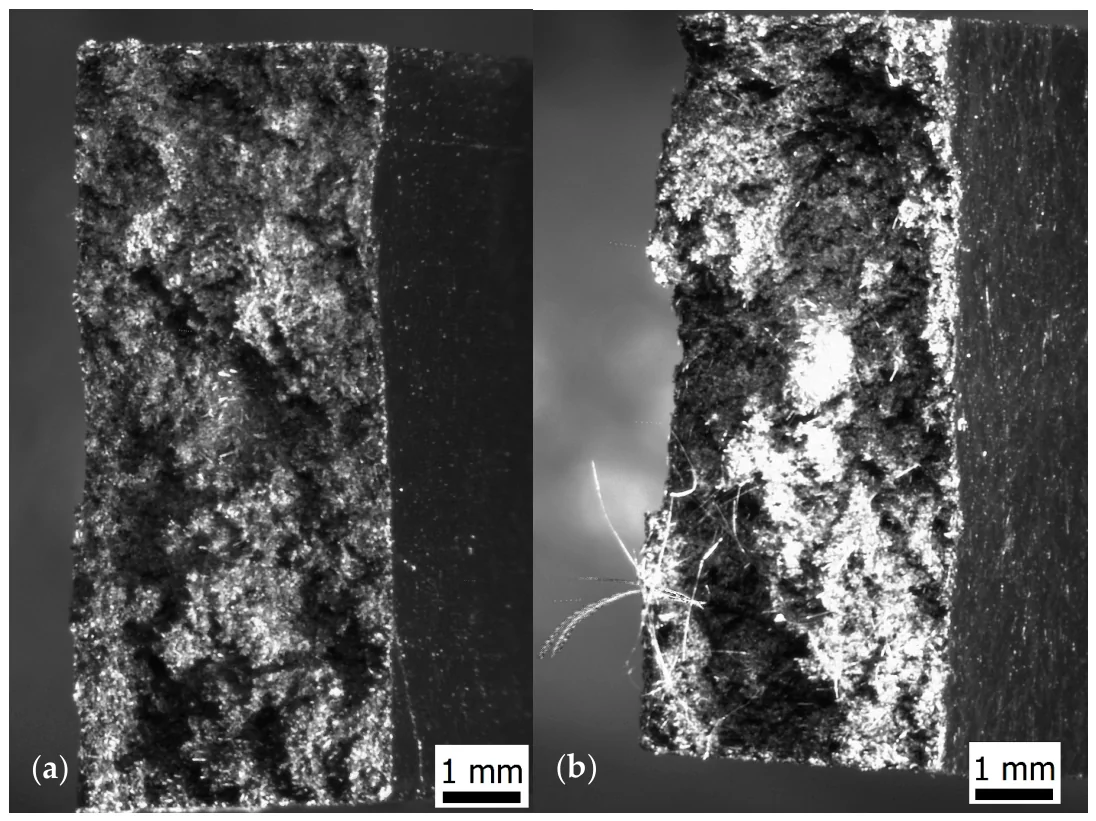
Representative fracture-surface morphology of selected PA66-GF25 tensile specimens: (**a**) initial dry reference specimen tested at 24 °C and (**b**) specimen thermo-oil-conditioned at 60 °C for 8 months and tested at 24 °C. Scale bars: 1 mm.

Representative fracture-surface images of selected PA66-GF25 specimens are shown in Figure 7. The images are included to provide qualitative visual documentation of the fracture appearance of the initial dry reference specimen and a specimen subjected to prolonged thermo-oil conditioning at elevated temperatures. In both selected conditions, the fracture surfaces exhibited features typical of short-glass-fibre-reinforced polyamide composites, including visible glass fibres, fibre pull-out and matrix fracture regions.

No quantitative fractographic evaluation was performed in the present study. Therefore, the fracture-surface observations are used only as qualitative supporting information and are not interpreted as direct evidence of the ageing mechanisms responsible for the observed tensile strength evolution.

## 4. Discussion

### 4.1. Possible Origins and Interpretation of the Non-Monotonic Behaviour

The present results revealed a non-monotonic evolution of tensile strength during thermo-oil conditioning. Specimens thermo-oil-conditioned at 24 °C and −30 °C exhibited an initial decrease in tensile strength after 4 months, followed by partial recovery and an apparent tendency towards stabilisation at longer exposure durations. Similar non-monotonic behaviour has been reported in studies dealing with environmental ageing of polyamide-based materials.

Because direct physicochemical and microstructural characterisation was not performed, the following discussion intentionally separates the experimentally observed macroscopic tensile response from possible mechanistic explanations. Several mechanisms may contribute to such behaviour. Previous studies have shown that fluid uptake can temporarily reduce mechanical properties through plasticisation of the polymer matrix, whereas longer exposure may be accompanied by structural relaxation, physical ageing, changes in crystallinity or other time-dependent processes affecting the mechanical response [3,5,7,8]. In fibre-reinforced composites, environmental exposure may also influence the fibre–matrix interface, thereby affecting stress transfer and failure behaviour [2,8].

The moisture state of the specimens may also have contributed to the observed tensile strength evolution. The initial non-immersed reference specimens were tested in the laboratory-conditioned state and were not additionally oven-dried before testing. Therefore, a certain amount of absorbed moisture may have been present in the initial reference specimens. Since moisture can plasticise polyamide matrices and reduce tensile strength, changes in moisture content during prolonged conditioning, particularly at 60 °C, may have contributed to the higher tensile strength measured after elevated-temperature conditioning. However, this effect could not be quantified because the moisture content of the specimens was not measured.

More generally, the present study did not include direct measurements of lubricant uptake, mass or dimensional changes, crystallinity, thermal transitions, viscoelastic behaviour or chemical/microstructural changes. Therefore, the individual contributions of moisture state, lubricant absorption, thermal exposure, structural relaxation, crystallinity changes and fibre–matrix interfacial effects cannot be separated on the basis of the available data. The mechanisms discussed above should therefore be regarded as possible explanations supported by the previous literature rather than experimentally confirmed mechanisms in the present study.

The observed recovery of tensile strength after prolonged exposure indicates that the material response cannot be described simply as progressive degradation. Instead, the results suggest that different ageing-related processes may dominate at different stages of thermo-oil conditioning. The non-immersed temperature-conditioned control specimens reported in the Supplementary Materials provide supporting qualitative reference data for the effect of thermal exposure without lubricant immersion. However, since only one valid specimen was available for each non-immersed control condition, these data were not used for formal statistical comparison.

Although the individual ageing mechanisms could not be separated, the tensile data obtained in the present study provide direct experimental evidence of a time-dependent macroscopic mechanical response of PA66-GF25 during long-term thermo-oil conditioning. In particular, the results demonstrate a non-monotonic tensile strength evolution at −30 °C and 24 °C and a continuously increasing trend after conditioning at 60 °C under the investigated conditions. The proposed mechanisms should therefore be interpreted as hypothesis-based explanations of these experimentally observed trends. Direct identification and quantification of the individual ageing mechanisms would require complementary physicochemical and microstructural analyses, such as DSC, FTIR, SEM and DMA.

Accordingly, the present tensile results demonstrate the macroscopic non-monotonic mechanical response, but they do not allow the individual contributions of lubricant uptake, moisture exchange, crystallinity evolution, chemical changes or fibre–matrix interfacial effects to be separated.

### 4.2. Effect of Conditioning Temperature

The conditioning temperature plays a key role in determining the mechanical behaviour of PA66-GF25. However, in the absence of direct physicochemical characterisation, the role of conditioning temperature can only be interpreted on the basis of the observed tensile response and trends reported in previous studies. Higher temperatures are generally associated with accelerated diffusion processes and increased molecular mobility in polyamide systems [4,5,7]. In addition, fluid conditioning of glass-fibre-reinforced PA66 has been shown to cause absorption, swelling or dimensional changes and modifications of mechanical performance [10,11].

Specimens thermo-oil-conditioned at 60 °C exhibited a continuous increase in tensile strength throughout the investigated exposure period. Similar behaviour has been reported in thermally aged glass-fibre-reinforced polyamides, where thermal ageing resulted in improved mechanical performance before degradation effects became dominant [14,15].

In contrast, for specimens thermo-oil-conditioned at lower temperatures (24 °C and −30 °C), such processes may occur more slowly, which could contribute to the more pronounced transient behaviour and delayed apparent stabilisation observed within the investigated time frame.

These observations indicate that conditioning temperature strongly influences the long-term evolution of tensile strength during thermo-oil conditioning.

### 4.3. Effect of Testing Temperature

The testing temperature had a pronounced influence on the measured tensile strength of PA66-GF25. For specimens thermo-oil-conditioned at 60 °C, tensile strength was substantially lower when tested at 60 °C than when tested at 24 °C. After 12 months of conditioning, tensile strength decreased from 165.2 ± 2.4 MPa when tested at 24 °C to 101.8 ± 4.1 MPa when tested at 60 °C.

Despite this reduction in absolute strength, the influence of thermo-oil conditioning remained evident under both testing conditions. Specimens thermo-oil-conditioned at 60 °C exhibited higher tensile strength than the corresponding initial dry reference condition tested at the same temperature. Furthermore, the increase in tensile strength with increasing exposure duration was observed for both testing conditions.

These findings indicate that thermo-oil conditioning and testing temperature influence different aspects of the mechanical response. While elevated testing temperature substantially reduced the measured tensile strength, the trends associated with long-term thermo-oil conditioning remained observable.

### 4.4. Engineering Implications

From an engineering perspective, the obtained tensile strength data are relevant as experimentally based input for the assessment of PA66-GF25 as a candidate material for polymer cage concepts in large-scale rolling bearings. Conventional cages for large bearing applications are commonly manufactured from steel. At large dimensions, their production may become costly and technologically demanding because of forming or machining operations, high material waste, long production times and high manufacturing costs associated with these production routes. For suitable cage geometries and production volumes, injection-moulded polymer cage concepts may provide a more cost-effective manufacturing route. Polymer cage concepts may therefore offer potential manufacturing and design advantages, but their use requires verification that the material retains sufficient mechanical performance under long-term exposure to bearing lubricant and service-relevant temperatures.

The present results are relevant to FEM-based assessment and optimisation of polymer cage concepts primarily as material-property selection and degradation-screening evidence. In practical simulations, manufacturer-provided material data are commonly used as input values for stiffness and strength. The one-year thermo-oil tensile results help to assess whether these baseline material properties need to be substantially reduced due to exposure-induced tensile-strength degradation after long-term contact with bearing lubricant and service-relevant temperatures.

A practical way of using the present tensile data in preliminary FEM-based screening is to treat the measured tensile strength ratios as conditioning-dependent strength-retention or degradation factors. For example, a tensile-strength retention factor can be defined as the ratio between the tensile strength after a given conditioning history and the corresponding reference tensile strength at the same testing temperature. Such a factor may be used to adjust allowable stress limits or safety margins in preliminary strength checks of polymer cage concepts. This approach does not replace a full constitutive material model, but it provides a conservative engineering screening tool for assessing whether long-term thermo-oil exposure causes a pronounced reduction in tensile strength.

The observed non-monotonic evolution is important for engineering assessment because short-term or intermediate exposure durations may not represent the long-term tensile response. In the present study, specimens thermo-oil-conditioned at 24 °C and −30 °C exhibited the lowest tensile strength after 4 months, followed by recovery and an apparent tendency towards stabilisation after longer exposure durations. This indicates that simplified extrapolation from a single intermediate ageing duration may lead to incomplete or misleading conclusions regarding long-term material behaviour.

Under the investigated conditions, no progressive deterioration of tensile strength was observed after 12 months of thermo-oil conditioning. This finding is relevant for preliminary FEM-based cage evaluation because it suggests that initial or manufacturer-provided material data may be used as a reasonable starting point for early-stage simulations, provided that appropriate safety factors, material state and service-temperature-dependent properties are considered. If a pronounced reduction in tensile strength had been observed after one year, a degraded material-property set would have been required for FEM-based screening.

The effect of testing temperature is also important for design interpretation. Although specimens thermo-oil-conditioned at 60 °C exhibited increased tensile strength when tested at 24 °C, their absolute tensile strength was substantially lower when tested at 60 °C. Therefore, material data used for design calculations or FEM-based assessment should correspond as closely as possible to the relevant service temperature range.

At the same time, the present results should not be interpreted as a complete validation of PA66-GF25 for long-term bearing cage operation. Tensile strength represents only one aspect of material performance. Confirmation of suitability for target service lives of 25−30 years would require additional long-term creep, fatigue, wear, dimensional stability, lubricant uptake, thermal cycling and component-level cage validation tests.

### 4.5. Limitations

The present study has several limitations that should be considered when interpreting the results. First, the temperature of the specimens during elevated-temperature and sub-ambient tensile testing was verified only prior to testing, while continuous temperature monitoring during loading was not available. Consequently, the reported testing temperatures should be regarded as nominal conditions established by the applied heating and cooling procedures.

Second, although up to five specimens were prepared for each condition, invalid failures occurring in the clamping region reduced the number of valid specimens included in the statistical evaluation. The number of valid specimens is therefore reported for each condition in Table 2. The reported results should be interpreted primarily in terms of overall trends rather than formal statistical significance.

In addition, tensile modulus and nominal strain-at-break were not evaluated as primary quantitative parameters because strain was obtained from crosshead displacement and extensometer measurements were available only for selected tests performed at 24 °C. Therefore, tensile strength was selected as the main consistently comparable mechanical parameter across all investigated conditions.

Furthermore, no direct measurements of lubricant uptake, mass change, dimensional change, crystallinity, thermal transitions, chemical changes, viscoelastic behaviour or microstructural changes were performed. Consequently, the mechanisms proposed to explain the observed non-monotonic evolution of tensile strength remain hypothetical and should be verified by complementary techniques such as DSC, FTIR, SEM and/or DMA in future work.

It should also be noted that complementary physicochemical and microstructural analyses should ideally be planned as part of the original experimental programme and performed on dedicated specimens immediately after removal from the conditioning environment. In the present study, such measurements were not performed at the time of specimen removal and tensile testing. Therefore, retrospective measurements on specimens after subsequent handling, testing or storage would not reliably represent the original post-conditioning material state. For this reason, these analyses are proposed as necessary future work to be performed on a newly prepared and controlled specimen series.

In addition, the moisture content of the initial reference and conditioned specimens was not measured. Therefore, possible changes in moisture state during long-term conditioning, especially at 60 °C, could not be separated from the effects of lubricant exposure and thermal ageing. This limitation should be considered when interpreting the increased tensile strength after elevated-temperature conditioning.

Future work should therefore combine long-term thermo-oil conditioning with complementary material characterisation. Mass uptake and dimensional measurements would allow the extent of lubricant absorption or swelling to be quantified. DSC analysis could be used to evaluate possible changes in crystallinity and thermal transitions, while FTIR spectroscopy could provide information about potential chemical changes in the polymer matrix after prolonged lubricant exposure. SEM observations of fracture surfaces and fibre–matrix interfaces would help to assess possible microstructural changes and interfacial degradation. In addition, DMA measurements would provide information about changes in viscoelastic behaviour and glass-transition-related response. Such complementary analyses would be necessary to verify the mechanisms responsible for the tensile strength evolution observed in the present study.

Finally, the present investigation focused exclusively on tensile behaviour. Additional studies addressing fatigue performance, creep resistance, wear behaviour, dimensional stability and component-level cage testing would provide a more comprehensive understanding of the long-term durability of PA66-GF25 under combined thermal and lubricant exposure conditions.

## 5. Conclusions

This study investigated the influence of long-term thermo-oil conditioning on the tensile behaviour of PA66-GF25 immersed in an industrial bearing lubricant at temperatures of −30 °C, 24 °C and 60 °C for periods of up to 12 months. The main scientific contribution of this work is the provision of a one-year experimental tensile dataset for PA66-GF25 under combined thermal and lubricant exposure conditions, which remains insufficiently documented in the available literature. The study also shows that the tensile response of PA66-GF25 under thermo-oil conditioning is not necessarily monotonic and cannot be reliably inferred from short-term or intermediate exposure data alone.

Based on the obtained results, the following conclusions can be drawn:

- The evolution of tensile strength during thermo-oil conditioning was non-monotonic for specimens conditioned at 24 °C and −30 °C. An initial decrease in tensile strength after 4 months was followed by recovery and an apparent tendency towards stabilisation at longer exposure durations.
- Specimens thermo-oil-conditioned at 60 °C exhibited a continuous increase in tensile strength throughout the investigated 12-month period, reaching the highest values within the main 24 °C testing series after prolonged exposure.
- The obtained results indicate that thermo-oil conditioning does not necessarily lead to continuous deterioration of tensile strength under the investigated conditions. Instead, the tensile response evolved through distinct stages depending on conditioning temperature and exposure duration.
- Elevated testing temperature substantially reduced the absolute tensile strength. Nevertheless, the relative trends associated with thermo-oil conditioning remained observable under both 24 °C and 60 °C testing conditions.
- The non-monotonic tensile response demonstrates that material properties determined after intermediate exposure durations may not be representative of long-term behaviour. This is relevant for service-oriented material assessment and FEM-based evaluation of polymer cage concepts, where material data representative of long-term thermo-lubricated exposure are needed.
- From an engineering perspective, the one-year thermo-oil tensile results support the further investigation of PA66-GF25 as a promising candidate material for polymer bearing cage concepts with long target service lives. However, confirmation of suitability for 25−30 years of operation requires additional long-term creep, fatigue, wear, dimensional stability and component-level validation tests.

The presented findings provide service-oriented tensile performance data for PA66-GF25 under combined thermal and lubricant exposure and can support material screening, FEM-based evaluation and durability assessment of polymer cage concepts. The results should not be interpreted as a direct prediction of bearing cage service life. Rather, the one-year conditioning period represents a practical long-term screening step for evaluating whether combined thermal and lubricant exposure causes pronounced degradation of PA66-GF25 tensile performance. Future work should focus on direct verification of the underlying ageing mechanisms using mass uptake and dimensional measurements, DSC, FTIR, SEM or DMA, together with longer-term exposure studies, fatigue, creep, wear and component-level cage validation.

## Figures and Tables

**Table 1:** Selected properties of Ultramid^®^ A3HG5 according to the manufacturer (BASF) [18].

Property	Unit	Value
Polymer type	-	PA66-GF25
Glass-fibre content	wt.%	25
Density	kg/m^3^	1320
Moisture absorption (23 °C/50% RH)	%	1.7–2.1
Water absorption (saturation, 23 °C)	%	5.7–6.3
Melting temperature (DSC)	°C	260
Tensile modulus (dry)	MPa	8500
Tensile modulus (conditioned)	MPa	5900
Tensile strength (dry)	MPa	175
Tensile strength (conditioned)	MPa	115
Strain at break (dry)	%	3.9
Strain at break (conditioned)	%	8.2

**Table 2:** Tensile strength statistics of initial dry reference and thermo-oil-conditioned PA66-GF25 specimens. The notation A/B denotes conditioning temperature (A) and testing temperature (B). For the 0 M dry reference, the indicated temperature refers only to the tensile testing temperature. Values are reported as mean ± standard deviation (SD). CV denotes the coefficient of variation.

Specimen Group	Exposure Time	Conditioning Temp.	Testing Temp.	n	Rm Mean ± SD (MPa)	CV (%)
Initial dry reference	0 M	none	24 °C	3	130.3 ± 3.8	2.9
Thermo-oil-conditioned	4 M	−30 °C	24 °C	3	119.0 ± 0.9	0.8
Thermo-oil-conditioned	8 M	−30 °C	24 °C	3	124.2 ± 0.5	0.4
Thermo-oil-conditioned	12 M	−30 °C	24 °C	3	125.2 ± 1.5	1.2
Thermo-oil-conditioned	4 M	24 °C	24 °C	3	118.0 ± 0.7	0.6
Thermo-oil-conditioned	8 M	24 °C	24 °C	3	125.3 ± 0.5	0.4
Thermo-oil-conditioned	12 M	24 °C	24 °C	3	126.4 ± 2.3	1.8
Thermo-oil-conditioned	4 M	60 °C	24 °C	3	145.8 ± 2.2	1.5
Thermo-oil-conditioned	8 M	60 °C	24 °C	3	159.8 ± 1.3	0.8
Thermo-oil-conditioned	12 M	60 °C	24 °C	3	165.2 ± 2.4	1.5
Initial dry reference	0 M	none	60 °C	2	72.8 ± 2.5	3.4
Thermo-oil-conditioned	4 M	60 °C	60 °C	3	82.7 ± 2.0	2.4
Thermo-oil-conditioned	8 M	60 °C	60 °C	3	91.9 ± 4.2	4.5
Thermo-oil-conditioned	12 M	60 °C	60 °C	3	101.8 ± 4.1	4.1

## Data Availability

The original contributions presented in this study are included in the article/Supplementary Material. Further inquiries can be directed to the corresponding author.

## Supplementary Materials

The following supporting information can be downloaded at https://www.mdpi.com/article/10.3390/polym18162008/s1. Table S1: Supplementary sub-ambient tensile strength results of initial dry reference and thermo-oil-conditioned PA66-GF25 specimens tested under nominal dry-ice cooling conditions; Table S2: Tensile strength values of non-immersed temperature-conditioned PA66-GF25 control specimens.
