# Supplementary material for: Long-Term Thermo-Oil Conditioning of PA66-GF25: Non-Monotonic Tensile Response Under Combined Thermal and Lubricant Exposure
Source: Polymers (Basel). 2026 Aug 18;18(16):2008. doi: 10.3390/polym18162008 (PMC13517394; doi:10.3390/polym18162008)
Supplement: Supplementary file 1 [file polymers-18-02008-s001.zip › polymers-4399666-supplementary.pdf]

Supplementary Material

Table S1. Supplementary sub-ambient tensile strength results of initial dry reference and thermo-oil-conditioned PA66-GF25 specimens tested under nominal dry-ice cooling conditions.

Specimen group	Exposure time	Conditioning temp.	Testing temp.	n	Rm mean ± SD (MPa)	CV (%)
Initial dry reference	0M	None	Nominal sub-ambient	2	161.1 ± 3.2	2.0
Thermo-oil-conditioned	4M	−30 °C	Nominal sub-ambient	3	185.3 ± 2.2	1.2
Thermo-oil-conditioned	8M	−30 °C	Nominal sub-ambient	3	182.5 ± 9.3	5.1
Thermo-oil-conditioned	12M	−30 °C	Nominal sub-ambient	3	190.1 ± 7.1	3.7

Note: The 0M condition denotes the initial non-immersed reference state without lubricant exposure or long-term thermal conditioning. The specimens were not additionally oven-dried before testing. Values are reported as mean ± standard deviation (SD). CV denotes the coefficient of variation. Low-temperature tensile experiments were performed using a custom insulated chamber with dry-ice cooling. A representative specimen exhibited a surface temperature of approximately −60 °C immediately before testing. Since the specimen temperature was not continuously monitored during loading, these results are reported as nominal sub-ambient data and are intended for qualitative comparison only.

Table S2. Tensile strength values of non-immersed temperature-conditioned PA66-GF25 control specimens.

Specimen group	Exposure time	Conditioning temp.	Testing temp.	n	Rm (MPa)
Non-immersed control	4M	24 °C	24 °C	1	119.6
Non-immersed control	4M	−30 °C	24 °C	1	119.2
Non-immersed control	4M	60 °C	24 °C	1	157.7
Non-immersed control	8M	24 °C	24 °C	1	127.8
Non-immersed control	8M	−30 °C	24 °C	1	123.6
Non-immersed control	8M	60 °C	24 °C	1	162.5
Non-immersed control	12M	24 °C	24 °C	1	134.2
Non-immersed control	12M	−30 °C	24 °C	1	123.2
Non-immersed control	12M	60 °C	24 °C	1	164.4

Non-immersed temperature-conditioned control specimens were stored for the same exposure duration and at the same conditioning temperature as the immersed specimens, but without lubricant exposure. Since one valid specimen was available for each condition, these values are reported as supporting qualitative reference data only.
